# Ecological Effect of Arginine on Oral Microbiota

**DOI:** 10.1038/s41598-017-07042-w

**Published:** 2017-08-03

**Authors:** Xin Zheng, Jinzhi He, Lin Wang, Shuangshuang Zhou, Xian Peng, Shi Huang, Liwei Zheng, Lei Cheng, Yuqing Hao, Jiyao Li, Jian Xu, Xin Xu, Xuedong Zhou

**Affiliations:** 1 State Key Laboratory of Oral Diseases, West China Hospital of Stomatology, Sichuan University, Chengdu, China; 2 Department of Operative Dentistry and Endodontics, West China Hospital of Stomatology, Sichuan University, Chengdu, China; 3 Department of Operative Dentistry and Endodontics, Hospital of Stomatology, Wenzhou Medical University, Wenzhou, China; 4 Single-Cell Center, Qingdao Institute of Bioenergy and Bioprocess Technology, Chinese Academy of Sciences, Qingdao, Shandong China; 5 Department of Pediatric Dentistry, West China Hospital of Stomatology, Sichuan University, Chengdu, China; 6 Department of Geriatric Dentistry, West China Hospital of Stomatology, Sichuan University, Chengdu, China

## Abstract

Dental caries is closely associated with the microbial dybiosis between acidogenic/aciduric pathogens and alkali-generating commensal bacteria colonized in the oral cavity. Our recent studies have shown that arginine may represent a promising anti-caries agent by modulating microbial composition in an *in vitro* consortium. However, the effect of arginine on the oral microbiota has yet to be comprehensively delineated in either clinical cohort or *in vitro* biofilm models that better represent the microbial diversity of oral cavity. Here, by employing a clinical cohort and a saliva-derived biofilm model, we demonstrated that arginine treatment could favorably modulate the oral microbiota of caries-active individuals. Specifically, treatment with arginine-containing dentifrice normalized the oral microbiota of caries-active individuals similar to that of caries-free controls in terms of microbial structure, abundance of typical species, enzymatic activities of glycolysis and alkali-generation related enzymes and their corresponding transcripts. Moreover, we found that combinatory use of arginine with fluoride could better enrich alkali-generating *Streptococcus sanguinis* and suppress acidogenic/aciduric *Streptococcus mutans*, and thus significantly retard the demineralizing capability of saliva-derived oral biofilm. Hence, we propose that fluoride and arginine have a potential synergistic effect in maintaining an eco-friendly oral microbial equilibrium in favor of better caries management.

## Introduction

Dental caries is one of the most prevalent infectious diseases worldwide^1, 2^, and is closely associated with the microbial disequilibrium between acidogenic/aciduric pathogens and alkali-generating commensal bacteria colonized in the oral cavity^3–6^. Novel strategies that suppress virulent species within a pathogenic biofilm could be effective alternatives to conventional antimicrobials that indiscriminately kill commensal bacteria^7–10^. Nourishing the dental plaque biofilm with substrates that encourage alkali production may inhibit tooth demineralization and favorably modulate the microbial metabolism and composition within dental plaque, thus being linked to the ecological management of dental caries^7, 11–13^. Metabolism of urea and arginine provide two major sources of alkali in dental biofilm^7, 13^. Arginine in the mouth is catabolized primarily by the microbial arginine deiminase system (ADS), which is presented in several streptococcal species, particularly *Streptococcus sanguinis* and *Streptococcus gordonii* ^7^. Previous work revealed a correlation between high salivary arginine concentration and caries resistance^14^. In addition, numerous cross-sectional studies found that caries-active or -experienced individuals exhibited significantly lower ADS activity compared with caries-free individuals^15–19^, further supporting the hypothesis that alkali-generation by oral bacteria may halt the development of dental caries.

Arginine has been incorporated into oral hygiene products for years. The Colgate-Palmolive Company has developed a Pro-Argin technology that contains 8% arginine in its toothpaste against tooth hypersensitivity^20^. In addition, the arginine-containing dentifrice has shown its potential in caries prevention. Oral hygiene products containing arginine bicarbonate (CaviStat^®^)^21, 22^ and toothpaste containing 1.5% arginine^23–27^, were proven to be highly effective against the initiation and progression of dental caries. The anti-caries effect of the arginine-containing dentifrice is attributed to its ecological effect on oral microbiota^10, 18^. In addition, a potential synergism between arginine and fluoride has also been indicated in our previous studies^10, 28^. The aim of the current study is to validate the microbiota-modulating effect of arginine with both clinical cohort and *ex vivo* oral biofilm model. We found that arginine treatment could modulate the oral microbiota of caries-active (CA) individuals to a consortium similar to that of caries-free (CF) individuals, characterized by enriched alkali-generating species and suppressed acidogenic species. In addition, the combinatory use of arginine with fluoride could synergistically suppress the demineralizing capability of saliva-derived oral biofilm.

## Results

### Treatment with arginine-containing dentifrice alters oral microbial composition

**Figure 1:**
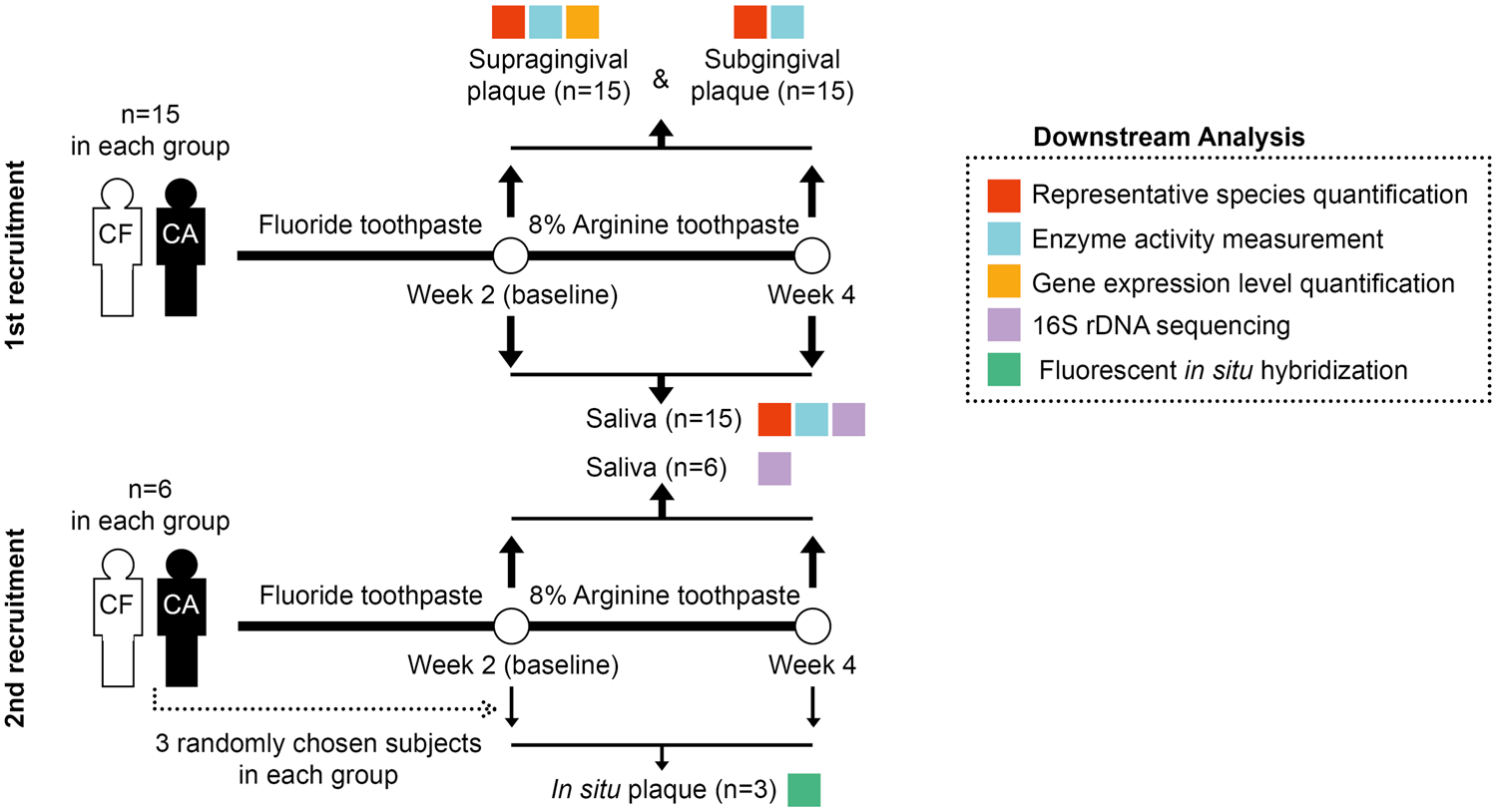
Scheme of the clinical study. N represents the sample size in each group. The different color blocks indicate the downstream analysis applied. CF: caries-free subjects; CA: caries-active subjects.

**Figure 2:**
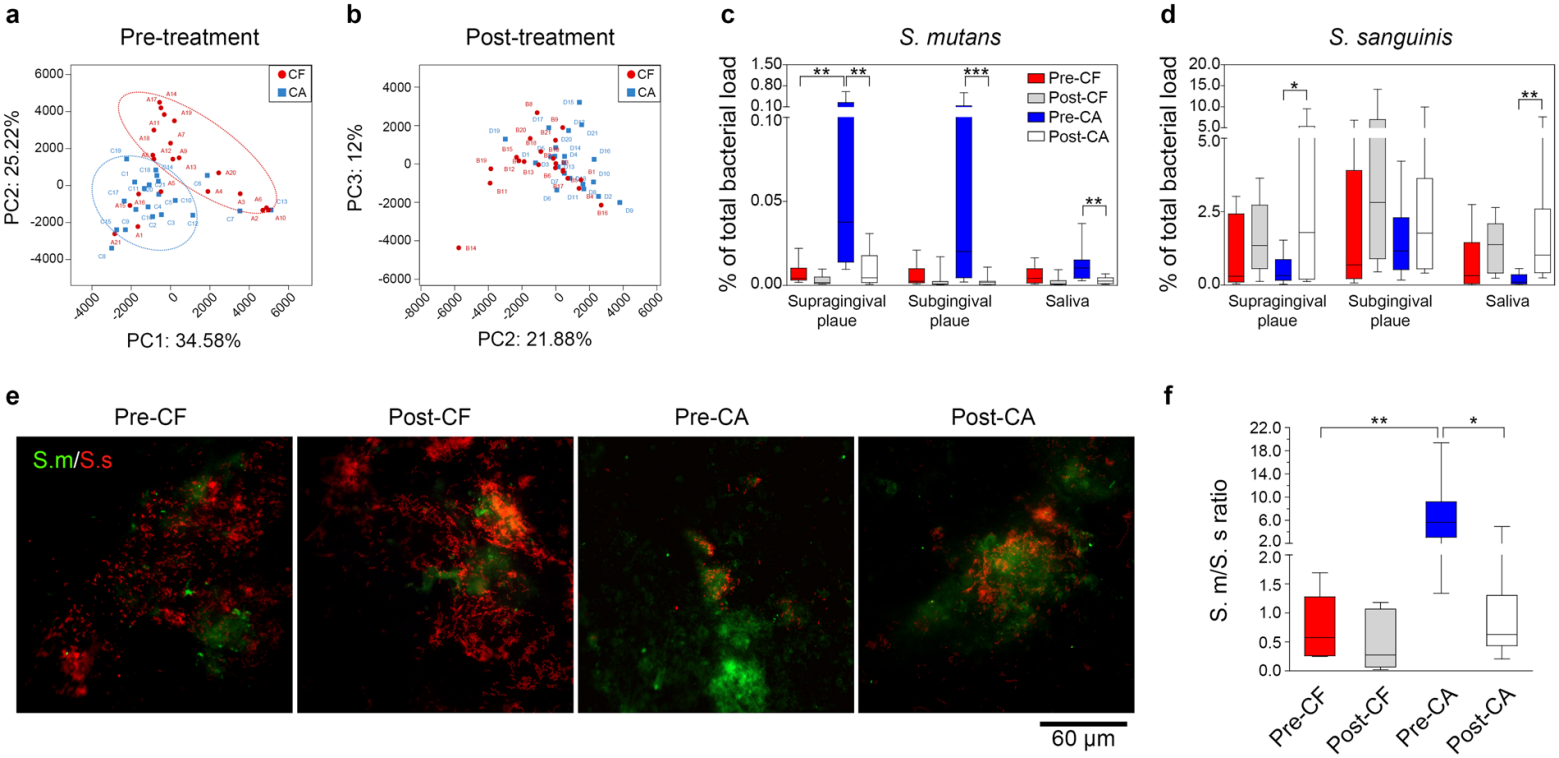
Treatment with arginine-containing dentifrice alters oral microbial composition. Principal Component Analysis (PCA) of saliva microbiome before (**a**) and after (**b**) 2-week arginine-containing toothpaste treatment (n = 21 in each group). Abundance of *S. mutans* (S. m) (**c**) and *S. sanguinis* (S. s) (**d**) in all types of samples before and after the treatment. Bacterial counts were determined by species-specific qPCR, and normalized with the total bacterial load. Data are presented as standard box plot, with the boxes presenting the first and third quartiles and the whiskers representing the 5th and 95th percentiles. (n = 15; Kruskal-Wallis test followed by Dunn’s multiple comparison test; *p < 0.05, **p < 0.01, ***p < 0.001). (**e**) Representative images of *in situ* plaques labeled by S. m- and S. s-specific fluorescent *in situ* hybridization (FISH) probe. (**f**) Quantitative analysis of S. m/S. s ratio in the *in situ* plaques, bacterial loads were measured based on integral optical density (IOD). Data are presented as standard box plot. (n = 3; Kruskal-Wallis test followed by Dunn’s multiple comparison test; **p < 0.05, **p < 0.01). Pre-CF and Post-CF = caries-free group before and after arginine-containing toothpaste treatment respectively; Pre-CA and Post-CA = caries-active group before and after arginine-containing toothpaste treatment respectively.

We recruited in total a cohort of 21 CF individuals with no clinical evidence of caries experience [decayed, missing and filled teeth (DMFT) = 0] and 21 CA individuals (DMFT ≥ 6) to investigate the effect of arginine-containing dentifrice on oral microbiota (Fig. 1 and Supplementary Table S1). Each individual was instructed to use 8% arginine toothpaste for two weeks. Saliva samples were collected before and after treatment, and microbial profiles were analyzed by bacterial 16S rDNA sequencing (n = 21 in each group). CA and CF groups exhibited distinct microbial community structure as demonstrated by Principal Component Analysis (PCA; Fig. 2a, Supplementary Table S2). After treatment, the salivary microbiome in both groups was more coherent (Fig. 2b, Supplementary Table S2).

We further examined the modification of microbial composition after the treatment. Operational Taxonomic Units (OTUs) classified at 3% dissimilarity were blasted with the oral “CORE” database, and relative abundance of each OTU was compared. We noticed that 4 OTUs (*i.e*., OTU_238, OTU_631, OTU_647 and OTU_398) belonging to the genus *Streptococcus* were repressed after arginine treatment (Supplementary Table S3). Species-specific qPCR was further used to investigate the effects of arginine on taxa belonging to the genus *Streptococcus*. We found that 2-week application of 8% arginine toothpaste reduced the abundance of *Streptococcus mutans* in supra-/sub-gingival plaque and saliva collected from the CA group (Fig. 2c). Meanwhile, the arginolytic species, *S. sanguinis* was enriched in supragingival plaque and saliva samples from CA group after treatment (Fig. 2d). The proportions of other arginolytic species (*i.e. S. gordonii*), a typical urealytic species (*Actinomyces naeslundii*) and a periodontal anaerobe (*Porphyromonas gingivalis*) were not altered (Supplementary Fig. S1).

Since the *S. mutans*/*S. sanguinis* ratio is believed to be correlated with dental caries^29, 30^, we further employed species-specific fluorescent *in situ* hybridization (FISH) to visualize the compositional alteration of these two bacteria in samples collected by a custom-designed *in situ* plaque collector (Fig. 2e, Supplementary Fig. S2). In parallel to the qPCR data, we observed that the *S. mutans*/*S. sanguinis* ratio was higher in the CA group (median = 5.10) relative to the CF group (median = 0.52; Fig. 2e and f) before the treatment, while the treatment with arginine-containing toothpaste substantially reversed this *S. mutans*/*S. sanguinis* disequilibrium in CA group (Fig. 2e and f).

### Treatment with arginine-containing dentifrice alters the enzymatic activity and expression of genes involved in the acid-base metabolism of oral microbiome

**Figure 3:**
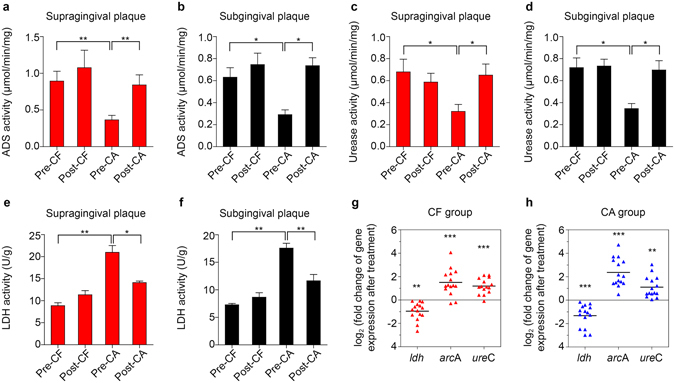
Treatment with arginine-containing dentifrice alters the enzymatic activity and expression of genes involved in microbial acid-base metabolism. Arginine deiminase system (ADS) (**a**,**b**), urease (**c**,**d**) and lactate dehydrogenase (LDH) (**e**,**f**) activities in supra- (**a**,**c**,**e**) and subgingival plaque (**b**,**d**,**f**). Data are presented as mean ± standard deviation (s.d.). (n = 15; one-way ANOVA test followed by Tukey’s test; *p < 0.05, **p < 0.01). The relative fold changes of *ldh*, *arcA* and *ureC* expression levels in supragingival plaques after treatment of every subject in caries free (CF) group (**g**) and caries-active (CA) group (**h**) are plotted. Data are presented as the results after log_2_ transformation, with the black horizontal lines representing the mean values (n = 15; paired sample t-test; **p < 0.01, ***p < 0.001). Pre-CF and Post-CF = caries-free group before and after arginine-containing toothpaste treatment respectively; Pre-CA and Post-CA = caries-active group before and after arginine-containing toothpaste treatment respectively.

Since the metabolic activities of oral bacteria are correlated with their cariogenicity^15–19, 31^, we holistically examined the acidogenicity, and arginolytic/urealytic activities of the oral microbiota. Microbiota obtained from CF individuals exhibited higher ADS and urease activities but a lower lactate dehydrogenase (LDH) activity relative to the CA individuals before the treatment (Fig. 3a–f). Treatment with arginine-containing dentifrice up-regulated the ADS/urease activities (Fig. 3a–d) and suppressed the LDH activities (Fig. 3e and f) in both supra- and sub-gingival plaques obtained from CA group, to an equivalent level of CF group after treatment. A similar trend was also observed in the saliva ADS activity of CA group, while the urease and LDH activities in saliva didn’t alter after treatment (Supplementary Fig. S3). Meanwhile, treatment with arginine-containing dentifrice did not alter the ADS/urease and LDH activities of plaque and saliva samples collected from CF group (Fig. 3a–f, Supplementary Fig. S3).

We further investigated the gene expression levels of *ldh* (LDH), *arc*A (arginine deiminase in ADS) and *ure*C (α-subunit of urease) in supragingival plaque samples (see primers in Supplementary Table S4). Consistent with observed changes in the enzymatic activities of supragingival plaque samples, *arc*A and *ure*C transcripts within the supragingival microbiome were up-regulated, and *ldh* was suppressed in CA individuals after arginine treatment (Fig. 3h). A similar trend in gene expression was also observed in the CF group (Fig. 3g), whereas no significant changes in corresponding enzyme activities were found (Fig. 3a,c and e). There were no significant differences between CF and CA group regarding the changes of *ldh*, *arc*A and *ure*C expression levels after the arginine toothpaste treatment.

### Arginine enriches *S. sanguinis* within dual-species biofilms

**Figure 4:**
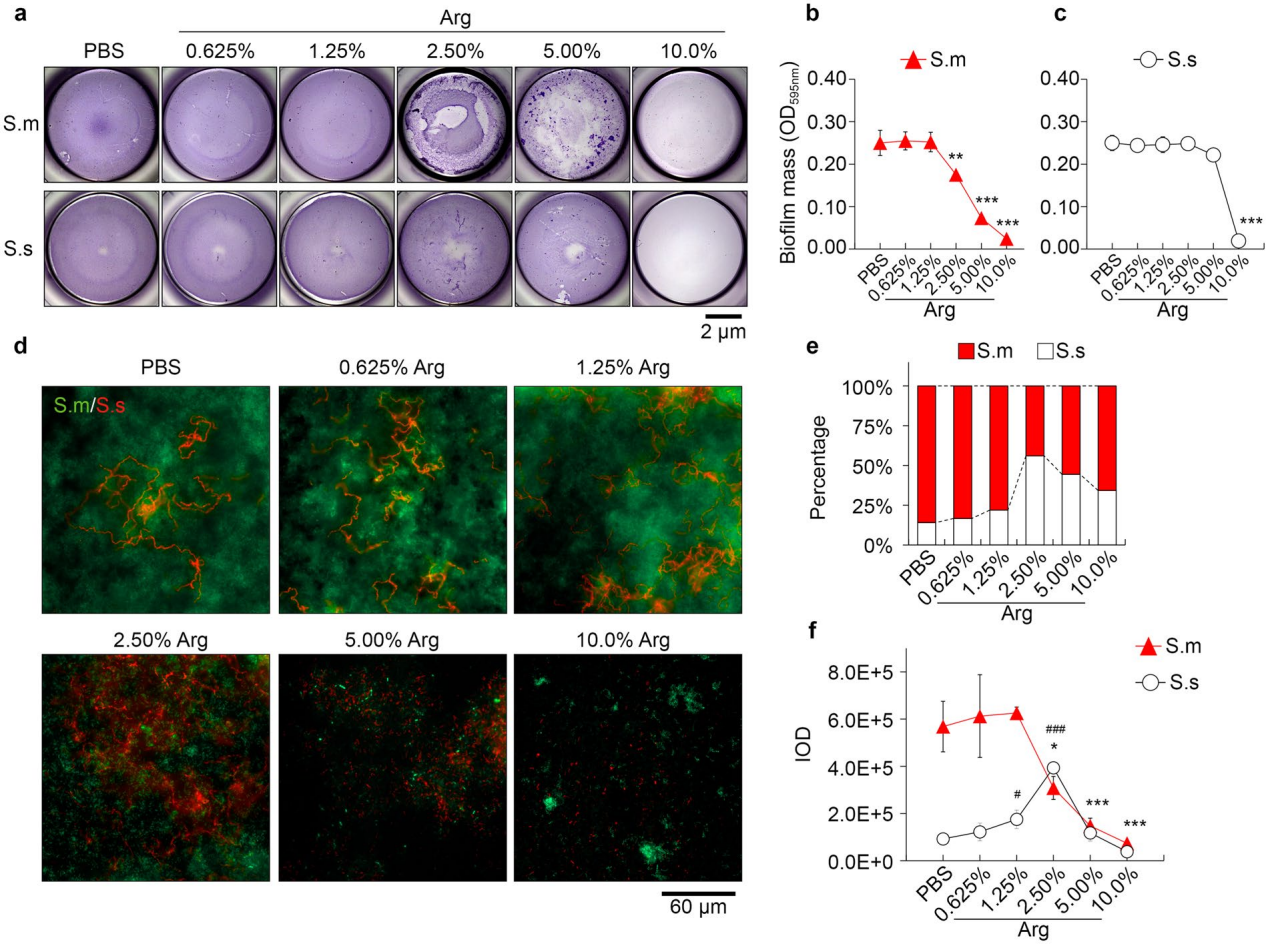
The destabilizing effect of arginine on *S. mutans* (S. m) and *S. sanguinis* (S. s) biofilms. (**a**) Representative stereomicroscope images of crystal violet stained 24-hour S. m/S. s biofilms treated with different concentrations of arginine (Arg). Quantitative analysis of S. m (**b**) and S. s (**c**) biofilms exposed to arginine. OD_595nm_ = optical density at 595 nm. Data are presented as mean ± s.d. (n = 3; one-way ANOVA test followed by Dunnett’s test to compare experimental groups with PBS-treated control group; **p < 0.01, ***p < 0.001). (**d**) Representative images of 24-hour dual-species biofilms labeled by S. m- and S. s-specific fluorescent *in situ* hybridization (FISH) probe. Quantitative analysis of S. m/S. s percentage (**e**) and number (**f**) based on integral optical density (IOD). Data are presented as mean ± s.d. (n = 3; one-way ANOVA test followed by Dunnett’s test to compare experimental groups with PBS-treated control group; significant difference in S. m number are indicated with *p < 0.05, ***p < 0.001, significant differences in S. s number are indicated with ^#^p < 0.05, ^###^p < 0.001).

Treatment with 2.5% arginine could inhibit the formation of *S. mutans* biofilms without suppressing bacterial growth, while 5% and 10% exerted more significant inhibition against both planktonic growth and biofilm formation of this acidogenic species (Fig. 4a and b; Supplementary Fig. S4). Nevertheless, the biofilm formation of *S. sanguinis* was almost unaffected by 0.625–5% arginine (Fig. 4a and c), indicating that the *S. mutans* biofilm was more vulnerable to high concentrations of arginine compared to *S. sanguinis* biofilm.

The inhibitory effect of arginine on biofilm formation was further investigated in the context of dual-species biofilms with species-specific FISH. Treatment with 0.625% and 1.25% arginine had no obvious effects on microbial composition within the biofilm (Fig. 4d–f). Interestingly, 2.5% and 5% arginine could reverse the *S. mutans*/*S. sanguinis* ratio in distinct manners (Fig. 4d–f). 2.5% arginine could suppress *S. mutans*, and increased the number of *S. sanguinis* in the dual-species biofilm compared to the PBS-treated control (Fig. 4d and f). On the other hand, 5% arginine could suppress *S. mutans*, but had no impact on the number of *S. sanguinis* compared to the PBS-treated control (Fig. 4d and f).

### Combinatory use of arginine augments the anti-demineralization effect of fluoride against saliva-derived biofilm

**Figure 5:**
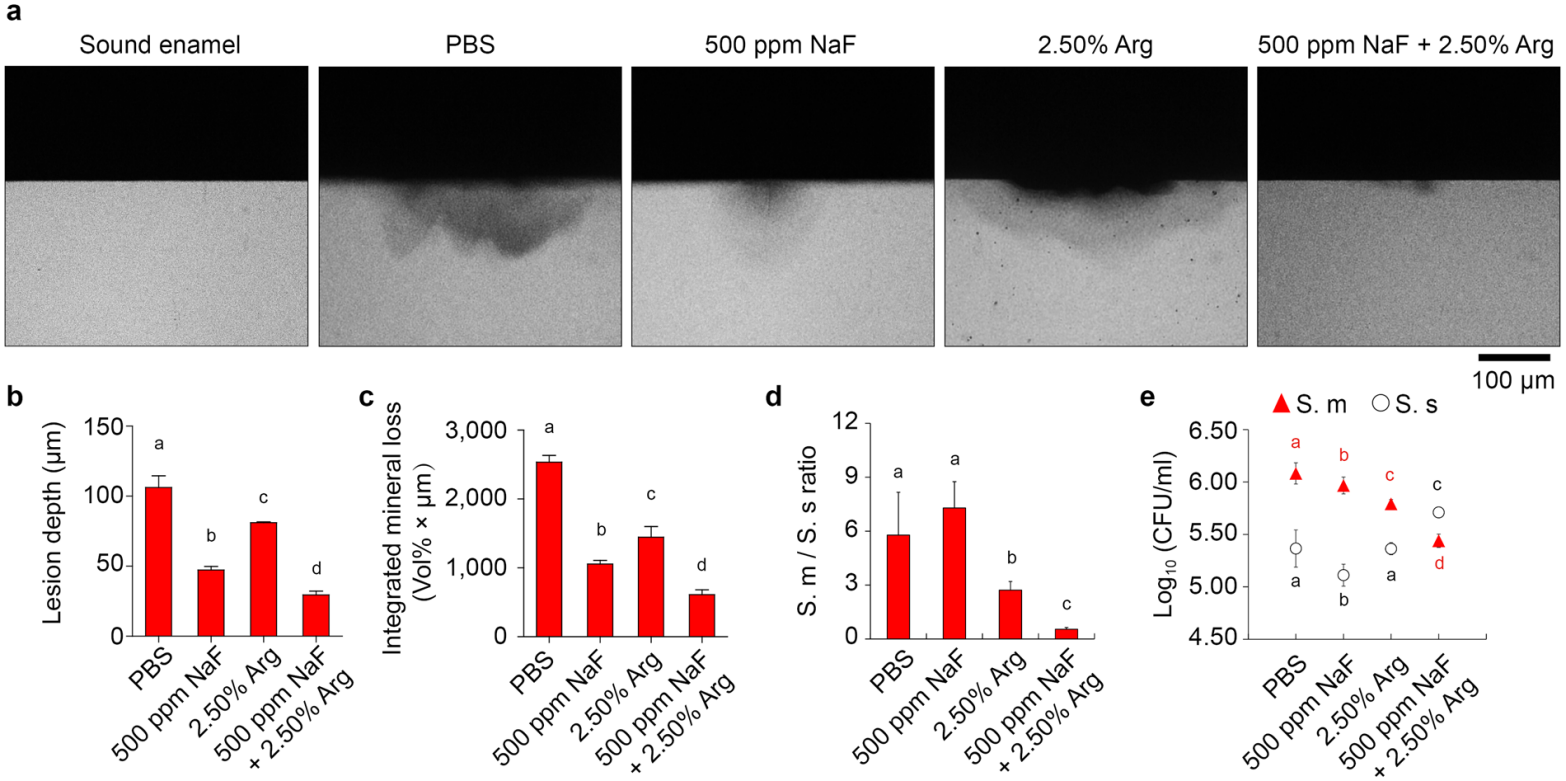
Combinatory use of fluoride augments the anti-demineralization effect of arginine against saliva-derived biofilm. (**a**) Representative Transverse Microradiography (TMR) images of human enamel discs exposed to 10-day biofilm-induced experimental demineralization. The high-density regions represent the sound enamel tissues, while the low-density shadows indicate the caries-like lesions. Lesion depth (**b**) and integrated mineral loss (**c**) were calculated. Data are presented as mean ± s.d. (n = 3; one-way ANOVA test followed by Student-Newman-Keuls test; different letters indicate significant inter-group differences, p < 0.05). Quantitative data of *S. mutans* (S. m)/*S. sanguinis* (S. s) ratio (**d**) and number (**e**) in the 24-hour human saliva-derived multispecies biofilm. Bacterial numbers were determined by qPCR. Data are presented as mean ± s.d. [n = 6; one-way ANOVA test followed by Student-Newman-Keuls test; different red letters (S. m number) and black letters (S. m/S. s ratio and S. s number) indicate significant inter-group differences, p < 0.05]. Arg = arginine; CFU = colony-forming unit.

Since most arginine-containing oral hygiene products also contain fluoride, we further test whether arginine could augment the inhibitory effect of fluoride against microbial demineralizing activity. We established a saliva-derived biofilm model, which better represent the diversity and overall metabolic functionality of human oral microbiome^8, 32, 33^, to evaluate the anti-demineralization effect of arginine alone or together with NaF. Biofilms grown on human enamel discs for 10 days, and were treated 3 times per day, with PBS, 2.5% arginine alone, 500 ppm NaF alone or 2.5% arginine and 500 ppm NaF in combination. Resulted lesions of enamel discs were examined with transverse microradiography (TMR). The severities of saliva-derived biofilm-induced lesions differed among different groups, with the arginine and NaF combinatory treatment exhibiting the least demineralization on the enamel surface (Fig. 5a–c).

Bacterial quantification by qPCR revealed that application of 2.5% arginine alone could reduce the *S. mutans*/*S. sanguinis* ratio in the saliva-derived biofilms (Fig. 5d), consistent with the data in dual-species biofilm model (Fig. 4d–f). Moreover, addition of NaF to the arginine treatment further decreased the *S. mutans*/*S. sanguinis* ratio by suppressing *S. mutans* and enriching *S. sanguinis* at the same time (Fig. 5d and e). Treatment of 500 ppm NaF alone repressed both bacteria in the biofilms (Fig. 5e), and have no impact on the *S. mutans*/*S. sanguinis* ratio (Fig. 5d).

## Discussion

Human tooth surfaces are colonized by hundreds of bacterial species. Bacteria with cariogenic potential are also frequently detected in healthy individuals^34–38^, suggesting an ecological etiology of dental caries. Frequent exposure of the microbial biofilm to carbohydrates leads to acid accumulation and subsequent pH declination, which selectively enrich acidogenic/aciduric species (e.g. mutans streptococci and lactobacilli) within biofilms and suppress those less aciduric commensal residents (e.g. *S. sanguinis*), and consequently drive the shift of microbial community to a more acidogenic/cariogenic consortium. This positive feedback mechanism leads to a continuous pH decline to the critical pH, below which tooth hard tissue demineralization begins and dental caries gradually occurs^4–6^. In the current study, we found that CF and CA subjects present distinct saliva microbiome, and CA individuals exhibited depressed alkali-generation capability, suggesting a microbial dysbiosis within cariogenic biofilms. Moreover, several metagenomic^39^, metatranscriptomic^40, 41^ and metaproteomic^42^ studies also showed that the oral microbiome of CA individuals exhibited distinct compositions and functions.

Due to the poly-microbial etiology of caries, an ecological management aiming to restore the microbial dysbiosis could be more promising. Arginine-containing oral hygiene products, which were previously used in the treatment of tooth hypersensitivity^20^, have been indicated to be effective in caries control^21–23, 43^. Its anti-caries effects have been attributed to its major impacts on the ecology of oral microbial communities by moderating the pH through ammonia production^7, 11, 13^. A similar study by Nascimento *et al*. found that, after 4-week treatment of 1.5% arginine dentifrice, the plaque microbial community of CA individuals and CF individuals became more coherent^18^. More recently, a study by Koopman *et al*. analyzed both salivary and plaque microbiomes of nine healthy individuals following 8-week application of 8% arginine toothpaste, and found a shift in the salivary microbial composition and function, but not in the plaque microbiome^44^. Here, we found that 2-week application of 8% arginine dentifrice favorably shifted the salivary microbiota of CA individuals to a community similar to that of CF individuals, characterized by increased alkali-generation and decreased acid production. These findings are consistent with the data reported by Nascimento *et al*.^18^. Interestingly, the *S. mutans*/*S. sanguinis* composition, as well as ADS, urease and LDH activities of dental plaque and saliva samples collected from CF group were unchanged after arginine treatment, indicating that arginine is likely to rescue a metabolic disequilibrium rather than to build a novel equilibrium of oral microbiome.

Although arginine is not commonly recognized as an antimicrobial agent, 8% arginine toothpaste could significantly suppress *S. mutans* biofilm formed on dentin discs^45^. Sustained millimolar concentrations of L-Arginine (>100 mM, about 1.7%) also reduced biofilm mass under flowing condition, and 500 mM of arginine even altered the microbial composition^46^. Though the mechanism of arginine being able to destabilize biofilm is not clear, previous data suggest that arginine may negatively influence the production of biofilm extracellular polysaccharides matrix^9, 47^, which plays a major role in the organization and cohesion of bacterial biofilms, especially that of *S. mutans* ^48^. In our *in vitro* dual-species biofilm assays, 2.5% of arginine remarkably down-regulated the *S. mutans*/*S. sanguinis* ratio, possibly due to the varied vulnerability of these two species to the biofilm-inhibition capability of arginine observed in the current study. Consistently, we also found 8% arginine treatment (approximately corresponding to a concentration of 2.67% arginine *in vitro*) significantly reduced the abundance of *S. mutans* (in supra-, subgingival plaque, saliva and *in situ* plaque samples) and enriched *S. sanguinis* (in supragingival plaque, saliva and *in situ* plaque samples) in CA individuals.

Most arginine-containing oral hygiene products also contain fluoride, a widely recognized anti-caries agent. Fluoride exerts its anti-caries effects mainly by improving the acid resistance of tooth hard tissue^49^. Recently, several clinical trials have shown that a dentifrice with arginine and fluoride could better prevent the initiation and progression of caries than fluoride alone^24–27, 50^. However, the mechanism of the synergism is yet unknown. In the current study, by utilizing a saliva-derived biofilm-induced caries model, we demonstrated that the superior anti-demineralization effect of the arginine/fluoride combination was possibly attributed to the synergism between these two agents in modulating the acidogenic/arginolytic species composition of biofilm. The observation that arginine and fluoride could synergistically modulate the microbial ecology of oral biofilm is consistent with our previous *in vitro* work^10^. In addition, our previous study also showed that arginine was able to promote fluoride uptake into artificial carious lesions, and subsequently enhanced the remineralization effect of fluoride on established lesions^28^. This may also partially explain the enhanced anti-caries effect of arginine and fluoride combination observed in the current study and other clinical studies.

Some cautions should be taken when interpreting data from this study. The lack of paralleled 16S rRNA sequencing data of plaque samples may hamper a comprehensive understanding of the ecological effect of arginine on the oral microbiome. Whether the benefits of arginine treatment were derived from specific reduction of *S. mutans*/*S. sanguinis* ratio or non-specific fix of a global dysbiosis still needs further study. Another limitation of the current study is that the enzymatic assays were carried out on frozen/thawed samples, which may not accurately reflect the instant enzymatic activity of the oral bacteria after arginine treatment.

Based on our current findings and previous data *in vitro* ^9, 10, 28^, we propose that arginine has a favorable ecological effect on oral microbiome, and thus represents a promising approach to caries control. Moreover, combinatory application of arginine and fluoride could synergistically attenuate enamel demineralization induced by saliva-derived multispecies biofilm, thus favoring better caries management.

## Methods

### Ethical Aspects

The study protocol was reviewed and approved by the Institutional Review Board of West China Hospital of Stomatology, Sichuan University (statement no. WCHSIRB-D-2013-067-R2), and was carried out in accordance with the guidelines. Written informed consent was obtained from all participants in the study. The trial was registered at the US National Institutes of Health (ClinicalTrials.gov) # NCT02988349, on November 24, 2016.

### Experimental Design and Sample Collection

21 CF individuals with no clinical evidence of caries experience (DMFT = 0) and 21 CA individuals (DMFT ≥ 6) were recruited (by 2 times) in the clinical trial (Fig. 1). The demographic data of the volunteers are listed in Supplementary Table S1. The exclusion criteria were applied: smoker or former smoker, presence of any systemic disease that could alter the production or composition of saliva, treatment with antibiotics, steroids or any medication known to cause dry mouth in the last 3 months, presence of dental prostheses or orthodontic devices, and presence of gingivitis or periodontitis. The clinical trial had a total duration of 4 weeks, comprised of washout phase of 2 weeks and a treatment phase of 2 weeks (Fig. 1). During the washout phase, subjects were instructed to brush their teeth twice daily for 3 min using Colgate^®^ Total^®^ toothpaste (containing 1450 ppm NaF), whereas during the treatment phase, subjects used Colgate^®^ Sensitive Pro-Relief^®^ toothpaste (containing 8% arginine and 1450 ppm NaF). Additionally, 3 randomly chosen subjects in each group in the second recruitment (see information in Supplementary Table S1) were asked to wear novel *in situ* plaque acquisition devices during both phases (patent no. 2014100507567; Supplementary Fig. S2; see details about the preparation of this device in Supplementary Information). The *in situ* plaque formed on hydroxyapatite discs (4 mm × 4 mm × 2 mm). Volunteers were asked to refrain from brushing and flossing their teeth, eating, and drinking anything other than water for 12 h prior to sample collection visits at the end of both phases. In the first recruitment, supra- and subgingival plaque (n = 15 in each group) and stimulated saliva (n = 15 in each group) samples were collected. In the second recruitment, stimulated saliva (n = 6 in each group) and *in situ* plaque samples (n = 3 in each group) were collected (Fig. 1). Scraped plaque was immediately transferred to and dispersed in sterile micro-centrifuge tubes containing 1 × PBS, and stored at −80 °C till further analysis. Saliva sample from each subject was evenly divided into 3 portions and stored at −80 °C. The *in situ* plaque samples were transferred into 4% paraformaldehyde, kept under 4 °C for 16 h, and then stored in 50% (v/v) ethanol at −20 °C.

### Salivary Microbiome Analysis

Genomic DNA of saliva samples (collected in the first and second recruitment, n = 21 from each group) were isolated using TIANamp bacteria DNA kit (Tiangen biotech, Beijing, China) according to the manufacturer’s instructions. The DNA quality was evaluated with a NanoDrop 2000 spectrophotometer (Thermo Fisher Scientific, Waltham, MA, USA), and final concentration was quantified via the Pico-Green kit (Invitrogen, Carlsbad, CA, USA). The barcoded 16S rRNA amplicon sequencing was performed through Illumina MiSeq technology where on average 330-bp-long reads were produced. Details on microbial 16S rRNA gene amplification, highly paralleled DNA sequencing, preprocessing of reads are included in the Supporting Information. The pre-processed sequencing data was further analyzed with statistical methods as following: (1) PCA was used to compare the phylogenetic structures between groups. Moreover, two non-parametric analyses for multivariate data, including analysis of similarities (ANOSIM)^51^ and non-parametric multivariate analysis of variance (Adonis) using distance matrices^52^ were used to examine the community difference between the two groups. (2) Taxonomic annotations were assigned to each OUT’s representative sequence by blasting with the oral “CORE” reference database^53^. The relative abundances of bacterial taxa at genus or species levels were analyzed using Student’s t-test.

### qPCR Analysis of Oral Samples

Genomic DNA of supragingival, subgingival and saliva samples (collected in the first recruitment, n = 15 from each group) were isolated using TIANamp bacteria DNA kit (Tiangen biotech) according to the manufacturer’s instructions. *S. mutans*, *S. sanguinis*, *S. gordonii*, *Actinomyces naeslundii*, *Porphyromonas gingivalis* and all bacterial counts were quantified using qPCR as previously described^54^ (see details in Supplementary Information; primers and probes were listed in Supplementary Table S4). Each sample was examined in triplicate.

### FISH

FISH was performed on the fixed *in situ* plaque samples (collected in the second recruitment, n = 3 from each group) as described previously^30^. Species-specific probes (Supplementary Table S4) were utilized to label *S. mutans* and *S. sanguinis* in the samples (see details in Supplementary Information). Pictures from at least 3 randomly selected positions of each labeled sample were captured using an Olympus BX3-CBH fluorescence microscope (Olympus Corp., Japan) equipped with an Andor iXon3 camera (Andor, Concord, MA, USA), the ISO (=400) and exposure time (=0.1S) were kept constant. Images were processed using Cell Sens Dimension (Olympus Corp.) without any qualitative changes to the raw images. The amount of bacteria was analyzed based on integral optical density (IOD) with Image pro plus 6.0 (Media Cybernetics, Silver Spring, MD, USA).

### Determination of Enzyme Activity of Microbial Samples

Enzyme activities were measured in supragingival, subgingival and saliva samples (collected in the first recruitment, n = 15 from each group). Urease and ADS activities were determined as described by Nascimento *et al*.^16^. In brief, ammonia generated from incubation (37 °C, 120 min) of 125 μl oral samples (suspended plaque and saliva samples) and a 500 μl mixture [50 mM urea (Sigma-Aldrich, St. Louis, MO, USA) or L-arginine-HCL (Sigma-Aldrich), 0.5 mM Tris-maleate buffer (pH = 6.0)] were measured by Nessler’s reagent (Sigma-Aldrich) with ammonium sulfate as a standard. Meanwhile, protein content in each sample was determined by Bradford’s Assay with bovine serum albumin as a standard. Urease and ADS activities were expressed as μmol ammonia produced per min and were normalized to mg of protein (μmol/min/mg). LDH activities in plaque and saliva samples were measured by the LDH Activity Assay Kit (Sigma-Aldrich) according to the manufacturer’s instructions. One unit of LDH was defined as the amount of the enzyme that catalyzed the conversion of lactate into pyruvate to generate 1.0 μmol of NADH per min. LDH activity was expressed as the amount of enzyme (U) per g of protein (U/g).

### Gene Expression Levels in Supragingival Plaque Samples

Total bacterial RNA from supragingival samples (collected in the first recruitment, n = 15 from each group) were isolated and purified according to the manufacturer’s instructions of RNeasy Mini Kit (Qiagen, Valencia, CA, USA), and reverse transcribed (500 ng RNA) using Prime Script RT Reagent Kit (TaKaRa, Japan) with random hexamer primers. qPCR amplification was performed on the CFX96 system (Bio-Rad, Hercules, CA, USA). The reaction mixture (25 μl) contained 1 × SYBR Premix Ex Taq (TaKaRa), 2 μl template cDNA, forward and reverse primers (500 nM each; sequences were listed in Supplementary Table S4). Expression level alterations of *arc*A, *ure*C and *ldh* after arginine-dentifrice treatment were calculated by Bio-Rad CFX Manager software (version 2.0) according to the 2^−∆∆ CT^ method.

### Bacterial Strains and Growth Media

*S. mutans* UA159 and *S. sanguinis* ATCC 10556 were commercially obtained from the American Type Culture Collection (ATCC). *S. mutans* and *S. sanguinis* were routinely grown at 37 °C under aerobic condition (5% CO_2_) in brain heart infusion broth (BHI; Difco, Sparks, MD). The potential inhibitory effects of L-arginine-HCL on the planktonic growth of *S. mutans* or *S. sanguinis* were monitored by measurement of the optical density of the cell culture at 600 nm (OD_600nm_). The effects of L-arginine-HCL on the biofilm formation of *S. mutans* or *S. sanguinis* were determined by crystal violet (CV) staining as described previously^55^. Each well of the 96-well microtiter plate contained overnight cultures of *S. mutans* or *S. sanguinis* (~1 × 10^6^ CFU/ml), 200 μl BHI containing 1% (m/v) sucrose, and L-arginine-HCL (0–10%). The images of CV stained 24 h biofilms were captured by EZ4-HD stereomicroscopy (Leica, Germany), and representative pictures were shown. The CV stain on the abiotic surface was de-stained with 95% ethanol and the biofilm biomass was determined by measuring the OD_595nm_ value of the collected corresponding de-stained solution. The data (normalized by the mean of non-treated control) are reported as the mean of 3 separate tests.

### *In vitro* Dual-Species Biofilm Model

Dual-species biofilm was established as described previously^10^. Briefly, overnight culture of *S. mutans* and *S. sanguinis* (~1 × 10^6^ CFU/mL each) were inoculated into 2 ml BHI containing 1% (w/v) sucrose and 0.625–10% (w/v) L-arginine-HCL (medium pH was adjusted to 7.0 prior to inoculation). Each well of the 24-well plate contained a piece of saliva-coated hydroxyapatite disc. The plate was incubated aerobically for 24 h. Then the biofilms on discs were labeled by species-specific FISH probes (listed in Supplementary Table S4). Biofilm images capturing, processing and bacteria quantification were performed as mentioned above.

### Human Saliva-Derived Multispecies Biofilm-Induced Caries Model

Multispecies biofilm was established by seeding pooled saliva (collected from 5 CA subjects in the current study, see information in Supplementary Table S1) into SHI medium, which is able to sustain the growth of an *in vitro* microbial community with a high diversity and similar microbial profiles to original salivary microflora^8, 32, 33^. Saliva-coated human enamel discs were prepared as described previously^28^ (see details in Supplementary Information), and were transferred aseptically into 24-well plate containing 30 μl pooled saliva and 1.5 ml SHI medium. Biofilms were let grow onto the discs under anaerobic conditions (90% N_2_, 5% CO_2_, 5% H_2_) at 37 °C for 24 h. Then discs with biofilm were exposed to PBS, 2.5% L-arginine-HCL alone, 500 ppm NaF alone or 2.5% L-arginine-HCL + 500 ppm NaF for 5 min three times per day (at 8:30, 12:30 and 16:30). To minimize the variation in baseline mineral level, enamel discs obtained from the same tooth were evenly distributed to each test group. The pH of all experimental solutions was adjusted to 7.0 prior to treatment. After exposure, specimens were washed with PBS and repositioned in the plate. SHI medium was refreshed after the third exposure in every day. After 10-day incubation, biofilms were detached and discs were washed. The result enamel discs were prepared as described by Eversole *et al*.^56^. X-ray films of experimental lesions were acquired by an X-ray generator (Softex, Japan) equipped with a microradiography camera, and then were further examined using Zeiss AXIO Imager A2 microscope (Carl Zeiss, Germany). Quantitative data was acquired by a calibrated analysis system TMR2006 (Inspektor Research Systems BV, Netherlands) (see details in Supplementary Information). Data are presented as the mean of 3 separate tests.

### Bacterial Quantification in Human Saliva-Derived Multispecies Biofilms

Aforementioned 24 h multispecies biofilms were scrapped using a sterile knife from saliva-coated human enamel discs into 1 ml PBS, sonicated to prepare the cell suspensions. Genomic DNA was isolated using a TIANamp bacteria DNA kit (Tiangen biotech) according to the manufacturer’s instruction. *S. mutans* and *S. sanguinis* numbers were determined by qPCR as mentioned above (see details in Supplementary Information; primers and probes used were listed in Supplementary Table S4). Data are presented as the mean of 6 separate tests.

### Statistical Analysis

Statistical analysis of data other than 16S rRNA sequencing was performed with SPSS (version 16.0 for Windows). qPCR and FISH quantitative data in clinical trial were analyzed by Kruskal-Wallis test to compare the means of all groups, and followed by Dunn’s multiple comparison test to test all pairs of groups. Gene expression data were analyzed by paired sample t-test. Other non-sequencing data were done with one-way ANOVA test, followed by Tukey’s test or Student-Newman-Keuls test to compare all pairs of groups, or Dunnett’s test to compare experimental groups with control group. Data were considered significantly different if the two-tailed p value was <0.05.

### Data Availability

The 16S rRNA sequencing raw data have been deposited in public database Sequence Read Archive (http://www.ncbi.nlm.nih.gov/Traces/sra) under accession number SRP082293.

### Clinical Trial Registration

The trial was registered at the US National Institutes of Health (ClinicalTrials.gov) # NCT02988349, on November 24, 2016.

## Electronic supplementary material

Supplementary Information
