# Supplementary material for: Ecological Effect of Arginine on Oral Microbiota
Source: Sci Rep. 2017 Aug 3;7:7206. doi: 10.1038/s41598-017-07042-w (PMC5543048; doi:10.1038/s41598-017-07042-w)
Supplement: Supplementary file 1 — Supplementary Information [file 41598_2017_7042_MOESM1_ESM.pdf]

Ecological Effect of Arginine on Oral Microbiota

Xin Zheng[1,2#], Jinzhi He[1,2#], Lin Wang[3], Shuangshuang Zhou[1,2], Xian Peng[1], Shi Huang[4], Liwei Zheng[1,5], Lei Cheng[1,2], Yuqing Hao[1,6], Jiyao Li[1,2], Jian Xu[4], Xin Xu[1, 2*], Xuedong Zhou[1,2*]

1. State Key Laboratory of Oral Diseases, West China Hospital of Stomatology, Sichuan University, Chengdu, China

2. Department of Operative Dentistry and Endodontics, West China Hospital of Stomatology, Sichuan University, Chengdu, China

3. Department of Operative Dentistry and Endodontics, Hospital of Stomatology, Wenzhou Medical University, Wenzhou, China.

4. Single-Cell Center, Qingdao Institute of Bioenergy and Bioprocess Technology, Chinese Academy of Sciences, Qingdao, Shandong, China

5. Department of Pediatric Dentistry, West China Hospital of Stomatology, Sichuan University, Chengdu, China

6. Department of Geriatric Dentistry, West China Hospital of Stomatology, Sichuan University, Chengdu, China

#These authors contributed equally to this work

*Corresponding authors: Xuedong Zhou, D.D.S., Ph.D., State Key Laboratory of Oral Diseases, West China Hospital of Stomatology, Sichuan University. No. 14, Section 3, Renmin South Road, Chengdu, China 610041. (O) 86-28-85501481; (email): zhouxd@scu.edu.cn. Xin Xu, D.D.S., Ph.D., State Key Laboratory of Oral Diseases, West China Hospital of Stomatology, Sichuan University. No. 14, Section 3, Renmin South Road, Chengdu, China 610041. (O) 86-28-85503494; (email): xin.xu@scu.edu.cn.

Supporting Information

***In situ* plaque acquisition device.** Custom-made *in situ* plaque acquisition palatal devices were made with six sites recessed into the polished surface (Supplementary Fig. S2). Hydroxyapatite discs (4 mm x 4 mm x 2 mm), which had been stored in 0.1% thymol solution (pH = 7.0) at 4 °C, were randomly assigned to each site and fixed with silicone rubber. Every site kept a 1 mm uniform gap covered by a plastic mesh to allow for the free contact of saliva with specimen's surface, and to protect it from mechanical disturbance. To minimize the contact between the tongue and the specimens, the sites were positioned posterior to the incisive papillae. Prior to the beginning of each phase, subjects had an appliance try-in appointment when needed adjustments were made. The participants wore the appliances more than 20 h every day allowing the salivary pellicle to have full access to the appliances in mouth, but withdraw them only during the main meals (3 times/day) and brushing time, when the appliance should be stored in humid conditions.

16S rRNA amplicon sequencing. The barcoded 16S rRNA amplicon (V1-V2 region) sequencing was performed through Illumina MiSeq technology at Majorbio, Shanghai. Primers used in present study was 27F (5'- AGAGTTTGATCCTGGCTCAG-3) and 338R (5'- TGCTGCCTCCCGTAGGAGT-3')[1]. A unique 12- mer tag for each DNA sample was added to the 5'-end of both primers to pool multiple samples for one run. PCR product was visualized on 3% agarose gels. Then, amplicons of all samples were gel purified, quantified with Pico-Green kit, pooled in an equal molar, assessed using Agilent BioAnalyzer 2100 (Invitrogen, USA), and sequenced. Sequences were trimmed using Trimmomatic [2] based on quality scores 20, and pair-end reads were merged into longer reads by FLASH [3]. Unqualified sequences were removed if they were too short or contained ambiguous residues. OTUs were clustered using Usearch（version 7.1, http://drive5.com/uparse/) at the 97% similarity level, and final OTUs were generated based the clustering results. The sequencing raw data has been deposited in public database Sequence Read Archive (http://www.ncbi.nlm.nih.gov/Traces/sra) with accession no. SRP082293.

Bacterial quantification by qPCR. qPCR amplification was performed on the CFX96 system (Bio-Rad, Hercules, CA). The reaction mixture (25 μl) contained Premix Ex Taq (TaKaRa, Japan), template DNA (100 ng), forward and reverse primers (500 nM each), and probes (250 nM). Thermal cycling conditions were designated as follows: initial denaturation at 95 °C for 30 s, followed by 40 cycles of 95 °C for 5 s and 56 °C for 30 s. Threshold cycle (CT) values were determined, and the CFU/ml was calculated based on the standard curve (Log CFU/ml versus CT) generated using standard strains respectively. *S. mutans*, *S. sanguinis*, *S. gordonii*, *A. naeslundii*, *P. gingivalis* and all bacteria were quantified (primers and probes were listed in Table. S4).

Fluorescence *in situ* hybridization. Fluorescence *in situ* hybridization was performed as described previously[4,5]. Fixed biofilms specimens established on hydroxyapatite discs were rinsed with distilled water and dried for 10 min at 46 °C. To enable probe penetration, the specimens were treated with 1 ml of lysis buffer [100 mM Tris-HCl (Sigma), 50 mM EDTA (Sigma), 30 mg/ml lysozyme (Sigma), pH = 8.0] for 20 min at 37 °C. The specimens were then rinsed with distilled water, serially dehydrated in ethanol (50%, 80% and 100%; 3 min each) and dried for 10 min at 46 °C. The specimens were then exposed to 20 μl of hybridization buffer (0.9 M NaCl, 20 mM Tris-HCl, 0.01% SDS, 20% formamide) containing the designated oligonucleotide probes (200 nM each) and incubated at 46 °C for 90 min in a closed cassette with a piece of paper-towel and 5 ml of hybridization buffer to equilibrate humidity. After hybridization, the glass slides were first washed in buffer (20 mM Tris-HCl, 5 mM EDTA, 0.01% SDS, 215 mM NaCl) for 15 min in a water bath at 48 °C, and then rinsed in ice-cold nuclease-free water. Custom synthesized oligonucleotide probes, labeled at the 5'-end with Alexa Fluor 488 or Alexa Fluor 594 were purchased from Invitrogen. Probe sequences are listed in Table. S4.

Biofilm images were examined using an Olympus BX3-CBH fluorescence microscope (Olympus Corp.) equipped with a 60× (1.42 numerical aperture) oil immersion objective lens, set SpGr-B Filter (Semrock, Inc.) for Alexa Fluor 488 and SpRed-B Filter (Semrock, Inc.) for Alexa Fluor 594. Black-and-white micrographs from at least 3 randomly selected positions of each sample were taken with an Andor iXon3 camera (Andor), the ISO (= 400) and exposure time (= 0.1s) were kept constant. Images were processed using Cell Sens Dimension (Olympus Corp.) without any qualitative changes to the raw images. The amount of bacteria was analyzed with Image pro plus 6.0 (Media Cybernetics, Silver Spring, MD) based on integral optical density (IOD). The data are reported as the mean of 3 separate tests, and representative pictures are shown.

Human enamel discs preparation. Twenty human permanent molars free of white spots, cracks and other defects were collected from the West China Hospital of Stomatology. The teeth were stored at 4 °C in water containing 0.05% thymol prior to sample preparation. Crowns were separated from roots and then cut into four sections (approximately 4 mm x 4 mm x 3 mm) with a diamond-coated saw (Struers Minitom; Struers, Denmark) under constant water cooling, resulting in an overall sample size of 80 enamel slabs. The enamel slabs were embedded in polymethylmethacrylate, and natural tooth surfaces were then polished progressively with water proof SiC abrasive papers (800-2400 grit; Struers), followed by polishing on a felt cloth impregnated with 1-5 μm diamond paste. This resulted in the removal of approximately 200 μm of the outer enamel layer. The specimens were ultrasonically cleaned in a deionized water bath for 2 min and then visually inspected to ensure removal of surface debris. All enamel discs were coated with one layer of acid-resistant nail varnish except for a rectangular window (3 x 2 mm) in the middle, and then disinfected with ultraviolet radiation. Portions of saliva samples collected from 5 CA

donors in the first recruitment (see information in Supplementary Table S1) were pooled together, diluted in PBS (1:5), and then filtered through 0.22 µm syringer syringe filter. The enamel discs were coated by immersing in aseptic human saliva under 37 °C for 2 h.

TMR analysis. After demineralization assay, **s**pecimens were longitudinally sectioned through the center using a hard tissue sectioning saw (Struers Minitom, Copenhagen, Denmark), and then polished progressively to thin plano-parallel sections (~100 µm thick) with water proof SiC abrasive papers (400-3000 grit). The sections were placed on a special designed Inspector sample-holder (Inspektor Research Systems BV, Netherlands) that was then fitted to a camera connected to an X-ray generator (Softex, Japan), and exposed to CuKα radiation. Developed X-ray films were examined using Zeiss AXIO Imager A2 microscope (Carl Zeiss, Germany) equipped with a digital camera. Acquired images were analyzed using TMR2006 (Inspektor Research Systems BV). In details, a "zero patch" and "sound patch" within the image will be identified automatically when import the image into the software. Then, a curve between mineral volume percentage (vol%) and sample position (µm) will be generated. Through this curve, lesion depth (µm) and integrated mineral loss (vol% × µm) will be calculated.

References

1 Ravel, J. *et al.* Vaginal microbiome of reproductive-age women. *Proc. Natl. Acad. Sci. U. S. A.* **108**, 4680-4687 (2011).

2 Bolger, A. M., Lohse, M. & Usadel, B. Trimmomatic: a flexible trimmer for Illumina sequence data. *Bioinformatics*, btu170 (2014).

3 Magoč, T. & Salzberg, S. L. FLASH: fast length adjustment of short reads to improve genome assemblies. *Bioinformatics* **27**, 2957-2963 (2011).

4 Zheng, X. *et al.* Involvement of gshAB in the interspecies competition within oral biofilm. *J. Dent. Res.* **92**, 819-824 (2013).

5 Klug, B. *et al.* Oral biofilm analysis of palatal expanders by fluorescence in-situ hybridization and confocal laser scanning microscopy. *J Vis Exp*, e2967-e2967 (2011).

Supplementary Fig. S1

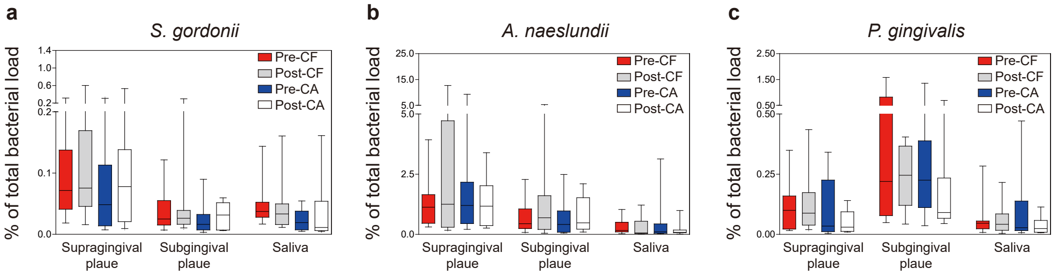

Supplementary Figure S1. Abundance of *S. gordonii* **(a)**, *A. naeslundii* **(b)** and *P. gingivalis* **(c)** before and after 2-week arginine-containing dentifrice treatment. Bacterial counts were determined by qPCR, and normalized with the total bacterial load. Data are present as standard box plot, with the boxes presenting the first and third quartiles and the whiskers representing the 5th and 95th percentiles. (n = 15; Kruskal-Wallis test). Pre-CF and Post-CF = caries-free group before and after arginine-containing toothpaste treatment respectively; Pre-CA and Post-CA = caries-active group before and after arginine-containing toothpaste treatment respectively.

Supplementary Fig. S2

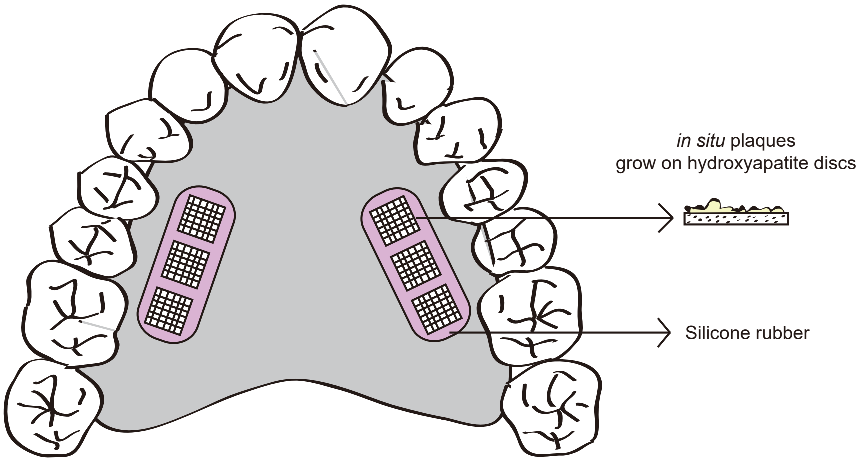

Supplementary Figure S2. Schematic diagram of customized *in situ* plaque acquisition device.

Supplementary Fig. S3

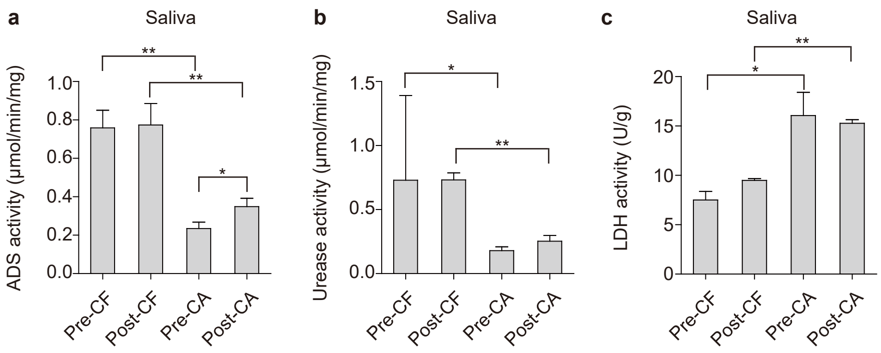

Supplementary Figure S3. Arginine deiminase system (ADS) **(a)**, urease **(b)** and lactate dehydrogenase (LDH) **(c)** activities in saliva samples before and after 2-week arginine-containing dentifrice treatment. Data are present as mean ± standard deviation (s.d.). (n = 15; one-way ANOVA test followed by Tukey's test; * $p < 0.05$, ** $p < 0.01$). Pre-CF and Post-CF = caries-free group before and after arginine-containing toothpaste treatment respectively; Pre-CA and Post-CA = caries-active group before and after arginine-containing toothpaste treatment respectively.

Supplementary Fig. S4

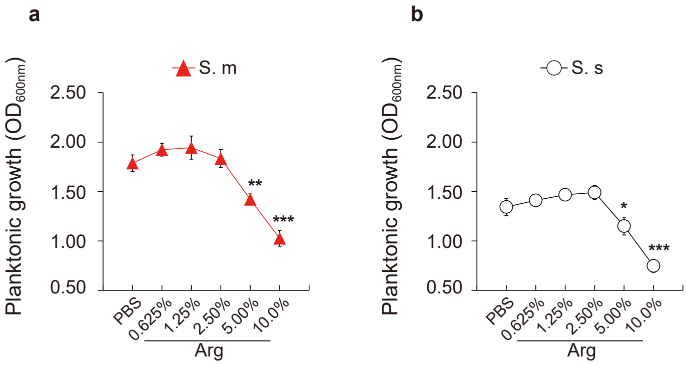

Supplementary Figure S4. 24-hour growth of *S. mutans* (S. m) **(a)** and *S. sanguinis* (S. s) **(b)** in planktonic cultures contained different concentrations of arginine (Arg). Data are present as mean ± s.d. (n = 3; one-way ANOVA test followed by Dunnett's test to compare experimental groups with PBS-treated control group; * $p < 0.05$, ** $p < 0.01$, *** $p < 0.001$). OD_{600nm} = optical density at 600nm.

Supplementary Table S1. Epidemiologic profile of the study group

CF: caries-free; CA: caries-active; DMFT: decayed, missing and filled teeth.
The saliva samples, from the 5 underscored individuals in CA group, were also used as inoculum in saliva-derived biofilm and for enamel discs coating.
The 3 asterisked individuals from each group in the 2nd recruitment were asked to wear the *in situ* plaque acquisition devices.

		Age	Gender	DMFT		Age	Gender	DMFT
1st recruitment	CF-1	21	M	0	CA-1	25	M	7
	CF-2	23	F	0	CA-2	27	F	7
	CF-3	24	F	0	CA-3	19	F	6
	CF-4	22	F	0	CA-4	23	F	9
	CF-5	21	M	0	CA-5	22	F	8
	CF-6	29	M	0	CA-6	28	F	7
	CF-7	20	F	0	CA-7	24	M	6
	CF-8	24	M	0	CA-8	20	F	6
	CF-9	24	F	0	CA-9	21	F	6
	CF-10	23	F	0	CA-10	19	M	6
	CF-11	23	F	0	CA-11	25	F	7
	CF-12	27	F	0	CA-12	25	F	6
	CF-13	19	F	0	CA-13	25	F	6
	CF-14	21	F	0	CA-14	19	F	7
	CF-15	27	M	0	CA-15	27	F	6
2nd recruitment	CF-16 *	21	M	0	CA-16	19	F	7
	CF-17 *	25	M	0	CA-17 *	20	F	8
	CF-18	26	F	0	CA-18	19	F	6
	CF-19	19	M	0	CA-19 *	22	F	7
	CF-20 *	31	M	0	CA-20	24	M	8
	CF-21	23	M	0	CA-21 *	23	F	7

Supplementary Table S2. Statistical analysis of saliva microbiota data

	ANOSIM		Adonis	
	R statistic	P value	R statistic	P value
Pre-CF v.s Pre-CA	0.094	0.027	0.05945	0.035
Post-CF v.s Post-CA	0.061	0.060	0.04623	0.065

Supplementary Table S3. Taxa with significantly different abundances between pre- and post- arginine exposure.

	OTU ID	Level	Phylogenic information		Post-treatment RA* (mean)	Pre-treatment RA (mean)	p value (Student's t-test)
**Caries active (16)**	OTU238	genus	*Streptococcus*	down	0.014428571427619	0.0272539682538095	0.0287322143279573
	OTU631	genus	*Streptococcus*	down	0.0179814814819048	0.0254550264557143	0.0406493412458229
	OTU647	genus	*Streptococcus*	down	0.00273809523857143	0.00436243386190476	0.00821312267871388
	OTU436	species	*Actinomyces_odontolyticus*	down	0.00192063492047619	0.00357671957714286	0.0190252673219292
	OTU635	species	*uncultured_Oribacterium_sp.*	down	0.000854497355714286	0.00161111111142857	0.0159943054817966
	OTU401	genus	*Granulicatella*	down	0.0010952380952381	0.00151851851857143	0.0629713714552705
	OTU59	species	*Capnocytophaga_leadbetteri*	down	0.000714285714285714	0.00142063492190476	0.103977546924764
	OTU278	species	*Prevotella_aurantiaca*	up	0.000888888889047619	0.000835978836666667	0.919644385534964
	OTU107	genus	*Neisseria*	up	0.0011058201052381	0.000637566137142857	0.0558813085065954
	OTU48	species	*uncultured_bacterium*	down	0.000314814816190476	0.000521164021428571	0.0805627814308618
	OTU576	species	*Capnocytophaga_sputigena*	down	0.000195767197142857	0.000481481480952381	0.00256717018032984
	OTU376	species	*uncultured_bacterium_oral_clone_BE109*	up	0.00100000000095238	0.000201058201428571	0.0618817295262861
	OTU474	species	*Capnocytophaga_sp._oral_taxon_326_str._F0382*	up	6080000000.0000	0.00012962963	0.0174168921911011
	OTU23	species	*Staphylococcus_epidermidis_RP62A_phage_SP.beta*	down	0.0000	2650000000.0000	0.0212335441002185
	OTU143	phylum	*Firmicutes*	down	582000000.0000	1590000000.0000	0.0997045248970978
	OTU422	species	*Eubacterium_brachy*	up	265000000.0000	18.5000	0.0300466474182891
**Caries free (22)**	OTU142	species	*uncultured_bacterium__g__Lautropia*	down	0.00319312169285714	0.00773015872904762	0.0333456756095828
	OTU677	species	*uncultured_bacterium__g__norank*	up	0.00718253968190476	0.00351322751285714	0.0634486356966597
	OTU101	species	*uncultured_bacterium__g__Porphyromonas*	up	0.00462433862428571	0.00194444444571429	0.0111908318032726
	OTU449	species	*uncultured_bacterium__g__Actinomyces*	up	0.00237566137571429	0.00106084656142857	0.00567353117475126
	OTU358	species	*uncultured_bacterium__g__Leptotrichia*	down	0.000500000000952381	0.000883597883809524	0.0241323053753485
	OTU228	species	*Prevotella_sp._oral_clone_ID019*	up	0.00236772486761905	0.000865079365238095	0.0944003504051492
	OTU344	species	*Prevotella_shahii*	up	0.000925925926666667	0.00046031746047619	0.0500469896422407
	OTU25	genus	*Actinomyces*	up	0.000722222223333333	0.000269841271428571	0.146035310883622
	OTU248	species	*Leptotrichia_trevisanii*	up	9259259333.3333	0.000251322751904762	0.143983565624927
	OTU242	species	*uncultured_candidate_division_TM7_bacterium*	up	105820109.5238	0.000187830688095238	0.123486361635921
	OTU607	species	*Kingella_denitrificans*	up	0.000534391534761905	0.000185185185714286	0.0190008932084693
	OTU619	family	*Enterobacteriaceae*	up	5291005285.7143	0.000164021164761905	0.0334255528805308
	OTU398	genus	*Streptococcus*	down	3174603190.4762	7407407428.5714	0.084158197632675
	OTU315	genus	*uncultured*	down	1851851952.3810	6613756714.2857	0.0203227408724832
	OTU378	species	*Clostridiales_bacterium_oral_taxon_093*	down	1587301619.0476	5820105857.1429	0.072476959760371
	OTU469	species	*Neisseria_sp._oral_clone_AP132*	down	0.000462962962857143	5555555571.4286	0.0257998650111579
	OTU166	species	*Leptotrichia_goodfellowii*	down	79365080.9524	4232804238.0952	0.0239341222456025
	OTU431	species	*uncultured_organism*	down	79365080.9524	2910052952.3810	0.0724769520712709
	OTU359	genus	*Acinetobacter*	down	0.0000	2116402190.4762	0.0422855865049289
	OTU231	species	*uncultured_actinobacterium*	down	0.0000	1058201142.8571	0.0422855890881888
	OTU655	genus	*Eremococcus*	down	0.0000	1058201142.8571	0.0422855890881888
	OTU395	species	*uncultured_bacterium*	up	2116402190.4762	0.0000	0.0422855865049289

* RA: relative abundance

Supplementary Table S4. Primers and probes used in the study

Primer or probe name	Sequence (5'-3')	Targets
smu_qPCR_f	GCCTACAGCTCAGAGATGCTATTCT	*S. mutans* *gtf*B
smu_qPCR_r	GCCATACACCACTCATGAATTGA	
smu_qPCR_p	FAM-TGGAAATGACGGTCGCCGTTATGAA-TAMRA	
san_qPCR_f	CTACCTTAGCACTTATCGTA	*S. sanguiinis* *gtf*P
san_qPCR_r	CTGTCCTGAACCACTATC	
san_qPCR_p	FAM-CTCTTGAACTGCCACCTGCT-TAMRA	
sg_qPCR_f	GGTGTTGTTTGACCCGTTCAG	*S. gordonii* *arc*A
sg_qPCR_r	AGTCCATCCCACGAGCACAG	
sg_qPCR_p	FAM-AACCTTGACCCGCTCATTACCAGCTAGTATG-TAMRA	
an_qPCR_f	ACGAAGACGCAAGGACAGAGG	*A. naeslundii* *ure*A
an_qPCR_r	GTAGGCCATGAGATCCGTGACC	
an_qPCR_p	FAM-CGCCTACATCACCGCTGAGATCCTCGA-TAMRA	
pg_qPCR_f	TACCCATCGTCGCCTTGGT	*P.gingivalis* *16S rRNA*
pg_qPCR_r	CGGACTAAAACCGCATACACTTG	
pg_qPCR_p	FAM-GCTAATGGGACGCATGCCTATCTTACAGCT-TAMRA	
universal_qpcr_f	CGCTAGTAATCGTGGATCAGAATG	*16S rRNA*
universal_qpcr_r	TGTGACGGGCGGTGTGTA	
universal_qpcr_p	FAM-CACGGTGAATACGTTCCCGGGC-TAMRA	
arcA_f	TTGCTAACAACCGTAAATTCATGC	*arc*A*
arcA_r	AAGATCGCCTTCGATTTCTGGGTGA	
ureC_f	CACGAGGACTGGGGTGCC	*ure*C**
ureC_r	CCCTTCGGTGTGGAAGGTATG	
ldh_f	GTTGCTGCTAACCCAGTTGACG	*ldh****
ldh_r	TCAGCAAGTGCTTGACGGAAA	
smu_fish	(Alexa fluor 488)-ACTCCAGACTTTCCTGAC	*S. mutans* *16S rRNA*
san_fish	(Alexa fluor 594)-GCATACTATGGTTAAGCCACAGCC	*S. sanguiinis* *16S rRNA*

*based on the *arc*A sequnces in *S. canis*, *S. cristatus*, *S. gordonii*, *S. ratti*, *S. sanguinis*, *S. suis*

** based on the *ure*C sequnces in *S. saliviarius, A. naeslundii, A. viscosus*

*** based on the *ldh* sequnces in *S. gordonii, S. mitis, S. mutans, S. pneumoniae, S. pyogenes, S. salivarius, S. sanguinis*
